# Gallotannin-Enriched Fraction from *Quercus infectoria* Galls as an Antioxidant and Inhibitory Agent against Human Glioblastoma Multiforme

**DOI:** 10.3390/plants10122581

**Published:** 2021-11-25

**Authors:** Nur Alisa Kamarudin, Nik Nur Hakimah Nik Salleh, Suat Cheng Tan

**Affiliations:** School of Health Sciences, Health Campus, Universiti Sains Malaysia, Kubang Kerian 16150, Kelantan, Malaysia; isa.addeen@gmail.com (N.A.K.); nikhakimahabdullah@yahoo.com (N.N.H.N.S.)

**Keywords:** medicinal plant, *Quercus infectoria* galls, gallotannin, glioblastoma multiforme, antioxidant, anticancer

## Abstract

In recent years, herbal medicine has experienced rapid development in the search for alternative anticancer compounds. Various phytochemicals present in *Quercus infectoria* (QI) galls have been reported to trigger cytotoxic effects on many types of cancer cells. However, a specific active constituent of QI galls with the potential to inhibit highly invasive stage IV malignant brain tumor, glioblastoma multiforme (GBM), is yet to be discovered. In this study, a two-phase system composed of aqueous soxhlet extraction and methanolic enrichment fractionation was employed to extract an anticancer compound, gallotannin, from the QI galls. This optimized two-phase system successfully generated a fraction (F4) with ~71% gallotannin, verified by the TLC and HPLC assays. Astoundingly, this fraction showed significantly higher (~1.15-fold) antioxidant activities compared to its crude extract, as well as to a commercial synthetic pure gallotannin. The F4 was also found to significantly suppress GBM cell growth, better than the synthetic pure gallotannin and the QI gall crude extract, probably related to its significantly higher antioxidant property. Moreover, the inhibitory effects exerted by the F4 treatment on GBM cells were comparable to the effects of two clinically used chemo-drugs (Temozolomide and Tamoxifen), indicating its high efficiency in combating human cancer. In conclusion, this study pioneered the development of an optimized extraction procedure for enriched yield of the natural gallotannin metabolite from the galls of the QI medicinal plant with high antioxidant potential and inhibitory effects on human GBM cells.

## 1. Introduction

Glioma is a broad category of tumors that occur primarily in the brain and spinal cord. It is the most prevalent type of adult brain tumor. Almost 80% of all types of malignant brain tumors and 30% of the central nervous system (CNS) tumors are categorized as glioma cancers [1]. Based on their location and presumed cell of origin, gliomas can be categorized into astrocytomas (derived from astrocytes, which provide nutrients and oxygen to the neurons in the brain), oligodendrogliomas (derived from oligodendrocytes, which insulate neurons with myelin sheath), ependymomas (derived from ependymal cells, which line the cavities in the brain) and mixed gliomas (a mixture of astrocytomas, oligodendrogliomas and ependymomas) [2].

Among all these types of gliomas, astrocytoma is the most commonly found glioma (representing about 75% of all gliomas) [1]. The World Health Organization (WHO) grading system is generally used to classify astrocytomas based on their specific histology features and malignant behavior. The grading includes Grade I: pilocytic astrocytoma, Grade II: diffuse astrocytoma, Grade III: anaplastic astrocytoma and Grade IV: glioblastoma multiforme (GBM) [2]. Currently, GBM is the most aggressive, invasive and undifferentiated type of astrocytoma. Standard therapies for GBM consist of maximal surgical resection, followed by radiotherapy and chemotherapy. The current standard clinical chemo-drug for GBM is Temozolomide, which is generally administered every day during radiation therapy and followed by six cycles after radiation during the maintenance phase [3]. Nonetheless, the response rate to the standard 5-day protocol for Temozolomide treatment is reportedly only 7–30% in GBM patients. In general, the median survival of GBM patients from initial diagnosis is less than 15 months, with a 2-year survival rate of 26–33% [4]. These data indicate that despite the availability of modern therapies for GBM, it is still a deadly disease with an extremely poor prognosis. Due to its high degree of malignancy and devastating outcomes, the development of alternative strategies to combat GBM is becoming a top concern in various ongoing scientific research, such as in the present study.

Recently, bioprospecting of anticancer active compounds from a wide array of herb plant species has offered valuable alternative resources for potential drug discovery. Natural product-based anticancer compounds are high in bioavailability and are cost-effective. Moreover, naturally derived compounds are generally more tolerated and non-toxic to normal human cells [5]. To date, the United States Food and Drug Administration (FDA) has approved multiple natural product-derived drugs to treat various forms of cancers. For example, the vinblastine and vincristine active compounds extracted from the *Catharanthus roseus* (*Apocynaceae*) and *Madagascar periwinkle* respectively, are currently used in clinical trials for leukemia, testicular cancer and other malignant tumors’ treatment [6,7]. In the present study, the anti-GBM therapeutic effect exerted by gallotannin, a potential active compound isolated from the gall of the *Quercus infectoria* (QI) plant, was investigated.

**Figure 1:**
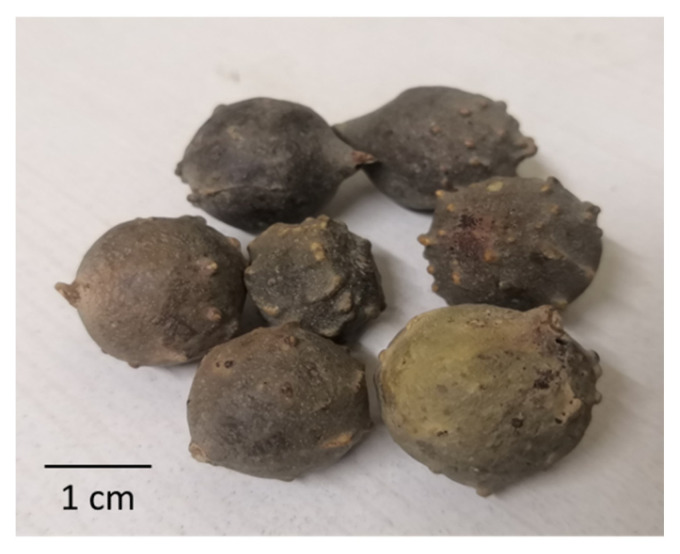
The galls of *Quercus infectoria* (QI) used in this study.

QI is an oak tree belonging to the Fagaceae (Quercaceae) family. Bioactive compounds can be found in various parts of the QI plant, including the bark, root, leaf, flower, seed, nut, legume and gall [8]. However, the main medicinal benefit of the QI plant lies in its gall, which is an excrescence formed due to the stimulus by Adleria gallae-tinctoriae gall-wasp egg deposition [9]. In Malaysia, the QI galls are commonly known as ‘manjakani’. The galls are usually spherical in shape, with a 1.0 to 2.5 cm diameter. The color is either grey, white-brown, olive green or dark bluish-green (Figure 1). Previously, our preliminary studies found that the QI galls contained a high concentration of gallotannin if subjected to soxhlet extraction using distilled water as a solvent [10].

Gallotannin is found as the major constituent (50–70%) in QI galls, present along with gallic acid (2–4%) and a small amount of free ellagic acid, starch and sugar [11]. Being the broadest phytochemical present in QI galls, gallotannin is strongly related to the biological effects induced by the QI galls, as reported previously [12,13]. Nonetheless, to date, there is still no study to report and validate the therapeutic potential of gallotannin extracted from the QI galls to treat the stage IV human brain tumor, GBM. Therefore, in this study, we aimed to extract the gallotannin from QI galls using a novel extraction procedure to determine its efficiency to treat GBM.

To achieve this aim, QI galls were subjected to dual-phase procedures consisted of soxhlet extraction using a water solvent and enrichment fractionation using an absorbent resin column flushed with gradient elution of a methanol-water solvent. The present study carefully optimized the extraction procedure for maximum recovery of good-quality bioactive gallotannin compound for biological assessments. Antioxidant and anti-GBM activities before and after the fractionation were evaluated to determine the potential of the gallotannin enrichment procedure developed in this study. Moreover, another notable significance of this study was the comparison of biological activities exerted by the naturally enriched gallotannin with a commercially available synthetic gallotannin, and two clinically used anticancer chemo-drugs, Temozolomide and Tamoxifen. This comparison could provide greater understanding of the differences in anticancer efficiency between natural products vs. synthetic origins or approved drugs.

## 2. Results

### 2.1. The Yield of Crude and Fractioned Extracts

From 50 g of QI gall powder, ~9.45 g (18.9%) of crude extract powder was obtained from aqueous extraction. In order to enrich the gallotannin active compound from QI galls, the harvested crude extract powder was fractionated through a Diaion HP-20 resin column eluted with methanol at gradient concentrations (0–100%). The elution of crude extract resulted in six methanol fractions (F1 to F6) with a complete recovery of the initial loaded material (7 g), which consisted of 2.24 g of F1 (0% methanol), 0.49 g of F2 (10% methanol), 0.63 g of F3 (25% methanol), 2.59 g of F4 (50% methanol), 0.98 g of F5 (75% methanol) and 0.07 g of F6 (100% methanol), respectively (Table 1).

### 2.2. Enrichment of Gallotannin Compound

**Figure 2:**
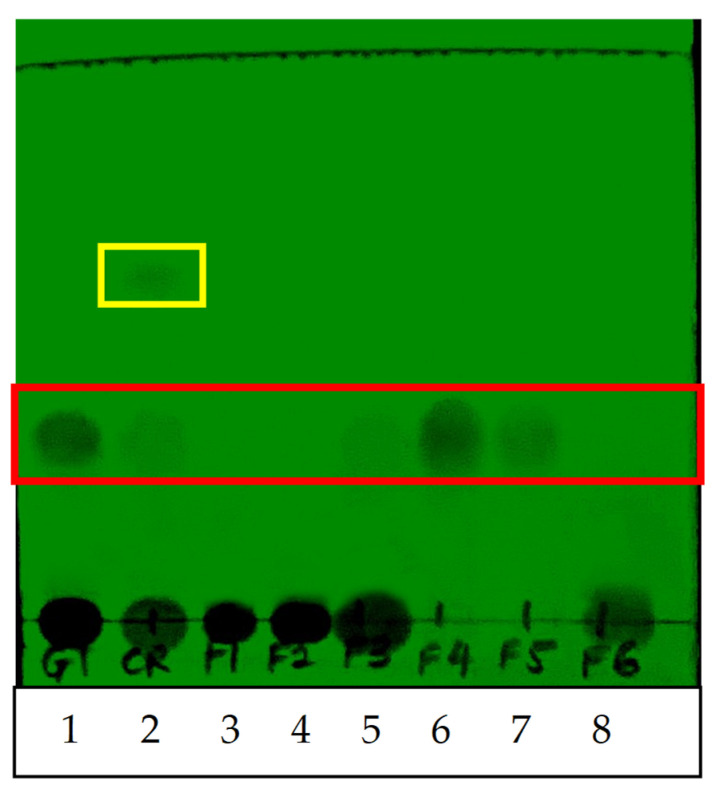
Thin-layer chromatography (TLC) plate analysis to detect the gallotannin compound using short UV wavelength (254 nm). Lane 1 = gallotannin synthetic compound (positive control); Lane 2 = QI gall crude aqueous extract; Lane 3 to Lane 8 = QI gall fractionated extracts F1 to F6. Red box indicates that gallotannin compound was detected in crude extract, F3, F4 and F5, while the yellow box indicates that an additional unknown compound was detected in the crude extract only.

QI crude and fractionated extracts (F1 to F6) were analyzed using thin-layer chromatography (TLC) to detect the gallotannin active compound (Figure 2). TLC plate screening under 254 nm ultraviolet light revealed the presence of the gallotannin compound (Lane 1, positive control) with the retention factor (Rf) value of ~3.14. Gallotannin was also spotted in QI gall crude aqueous extract (Lane 2), F3 (Lane 5), F4 (Lane 6) and F5 (Lane 7). The identity of gallotannin in these sample was verified because the bands detected in these samples had the same Rf value as the standard gallotannin control (Figure 2, indicated by the red box). Moreover, based on the band intensity, it was estimated that gallotannin from the crude extract was abundantly retained in F4, followed by F3 and F5, after the fractionation procedure. On the other hand, the earlier fractions (F1–F2, Lane 3–4) and last fraction (F6, Lane 8) did not show any clear spot of gallotannin, indicating that gallotannin was absent/minimal in these fractions. Besides, an additional unknown compound was also detected in the crude extract with an Rf value of ~0.60 (Figure 2, indicated by the yellow box). However, this additional compound was not detected in other fractionated samples (F1 to F6), indicating that the purification of the crude extract sample to isolate the targeted gallotannin compound using the dual-phase enrichment procedure described in this study was successful.

**Figure 3:**
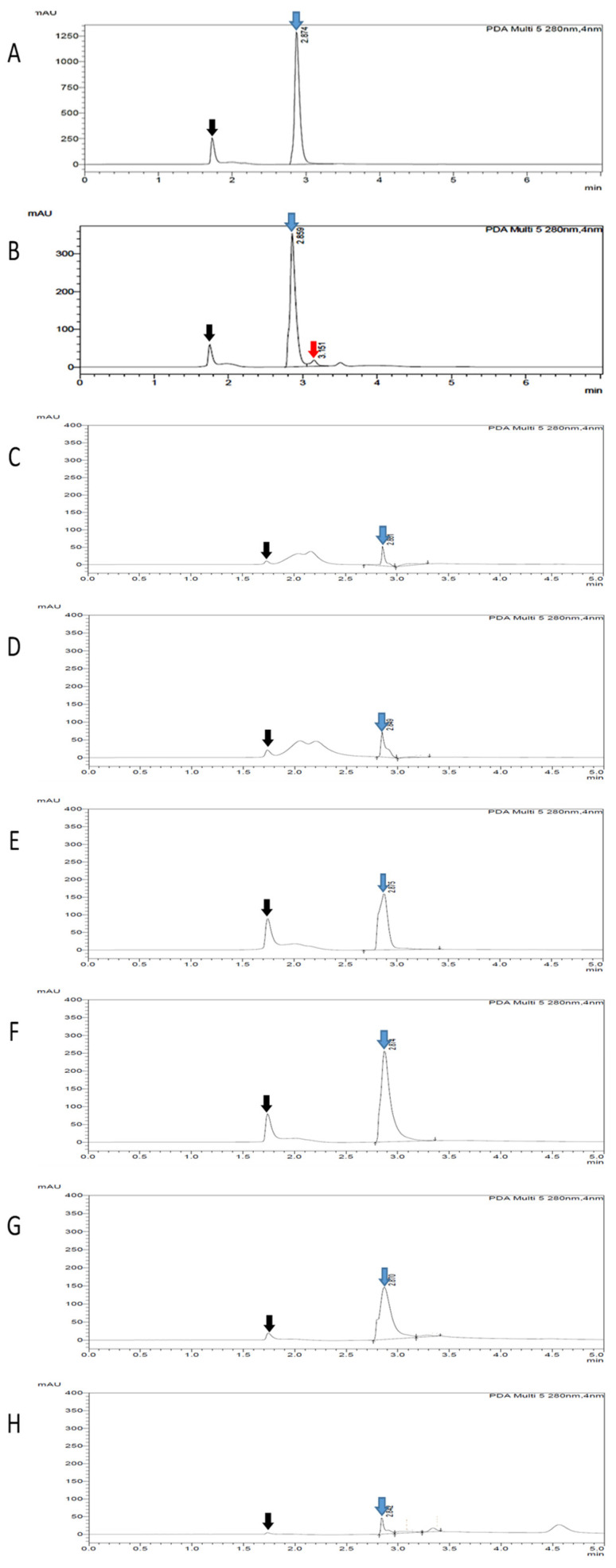
High-performance liquid chromatography (HPLC) analysis to detect and quantify the gallotannin compound. (**A**) Synthetic pure gallotannin (positive control), (**B**) QI crude aqueous extract, (**C**) F1, (**D**) F2, (**E**) F3, (**F**) F4, (**G**) F5 and (**H**) F6. Blue arrows indicate that gallotannin compounds were detected at a retention time of ~2.874 min. Red arrow indicates an extra unknown compound detected at a retention time of ~3.151 min in the QI crude extract only. Black arrows indicate that a prominent peak appeared before the gallotannin due to the oxidation of samples during processing of samples.

**Figure 4:**
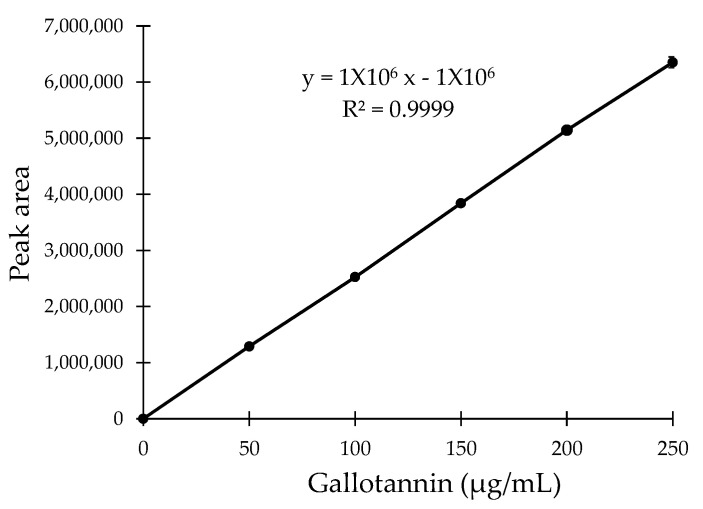
Standard calibration curve to determine the concentration of gallotannin in the fractionated QI extract (F1 to F6).

**Figure 5:**
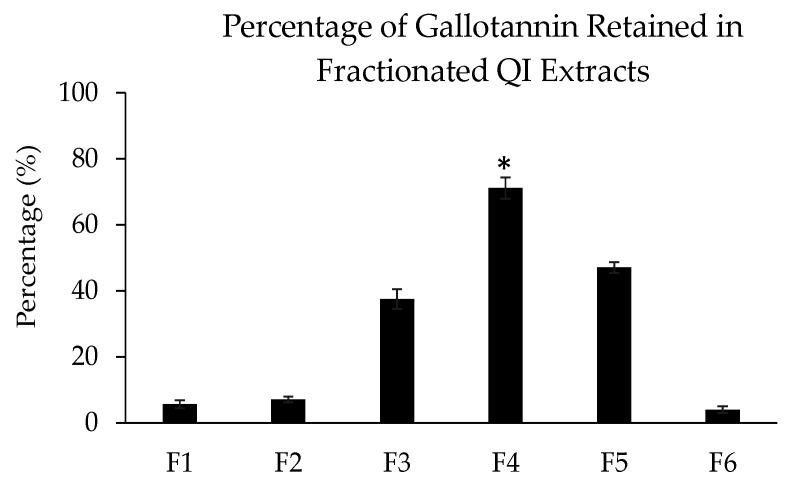
Percentage of the gallotannin compound retained in the fractionated QI extract (F1 to F6) (* *p* < 0.05 for F4 vs. all other fractions).

The high-performance liquid chromatography (HPLC) assay was conducted to verify the TLC result. Based on the HPLC analysis, the gallotannin was eluted at the retention time of ~2.874 min (Figure 3A–H, indicated by blue arrows). A prominent peak that appeared before the gallotannin peaks might correspond to the oxidation of gallotannin during the preparation process of samples for HPLC analysis (Figure 3A–H, indicated by black arrows). Moreover, similar to the TLC plate result, an extra unknown compound with a retention time of ~3.151 min was detected in the QI crude extract (Figure 3B, indicated by the red arrow). However, this additional compound was not detected in synthetic pure gallotannin or other fractionated extracts. This data, again, suggested that the fractionation procedure described in this study had successfully refined non-targeted compounds from the crude extract, generating an enriched extract of gallotannin. After the HPLC assay, a calibration curve was plotted using the standard gallotannin compound at gradient concentrations (*x*-axis) against the peak area (*y*-axis) (Figure 4). The plotted curve was linear with a 0.9998 correlation efficiency, indicating that it was accurate and reliable. Based on this standard gallotannin calibration curve analysis, the amount of gallotannin in each fraction was calculated. It was found that F4 significantly yielded the highest amount of gallotannin (71.15 ± 3.21 µg/mL) compared to all other fractions (Table 2 and Figure 5). Therefore, F4 was selected to be investigated in all subsequent experiments.

### 2.3. Antioxidant Potential

#### 2.3.1. DPPH Free Radical Scavenging Activity Assay

**Figure 6:**
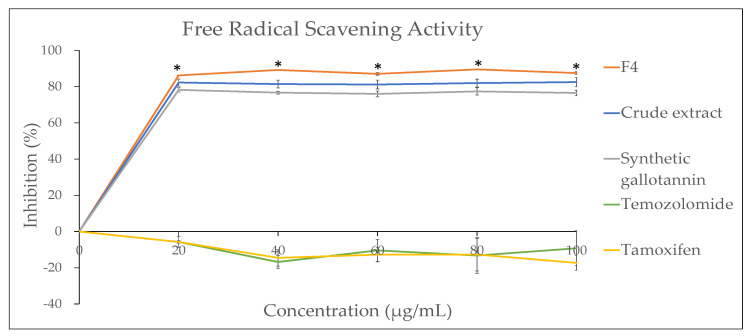
Free radical scavenging activity analysis for QI gall extract before fractionation (crude extract) and after fractionation (F4), along with the synthetic gallotannin compound and chemo-drugs (Temozolomide and Tamoxifen) (* *p* < 0.05 for F4 vs. all other groups).

In order to evaluate the antioxidant potential of QI galls before fractionation (crude extract) and after fractionation (F4), the DPPH (2,2-diphenyl-1-picryl-hydrazyl-hydrate) assay was performed along with synthetic pure gallotannin as a positive control. It was found that F4 showed significantly higher free radical scavenging activities compared to the QI crude aqueous extract, as well as the synthetic pure gallotannin from the lowest concentration (20 µg/mL) until the highest concentration (100 µg/mL) (Figure 6). This indicated that the gallotannin enriched from the QI gall crude extract was highly effective as an antioxidant agent compared to the crude extract, as well as the synthetic gallotannin compound. In addition, the antioxidant properties of two clinically used chemo-drugs (Temozolomide and Tamoxifen) were also determined in the present study. Notably, it was found that both chemo-drugs showed no antioxidant activity at all. Moreover, at higher concentrations (40–100 µg/mL), both chemo-drugs were found to increase the free radical percentage. The data are in line with a previous publication which stated that chemotherapy could be an indirect source of generating free radicals, resulting in oxidative damage [14].

#### 2.3.2. Reducing Power Assay 

**Figure 7:**
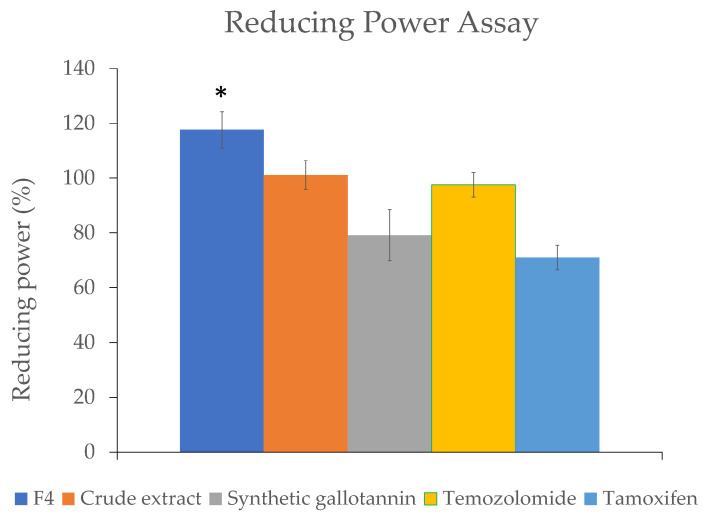
Reducing power assay for the QI gall extract before fractionation (crude extract) and after fractionation (F4), along with the synthetic gallotannin compound and chemo-drugs (Temozolomide and Tamoxifen) (* *p* < 0.05 for F4 vs. all other groups).

As well as the DPPH assay, we also performed the reducing power assay to compare the antioxidant activity of gallotannin-enriched F4 to its crude extract, synthetic gallotannin and chemo-drugs (Temozolomide and Tamoxifen). The reducing capacity of a compound may serve as a significant indicator of its potential antioxidant activity. A higher reducing power indicates higher antioxidant potential. Similar to the DPPH assay data, the reducing power of F4 was found to be significantly higher than all other groups, indicating that the fractionation procedure described in this study significantly enhanced the antioxidant property of the QI gall extract (Figure 7).

### 2.4. Cytotoxicity Effects on Human GBM Cancer Cells

**Figure 8:**
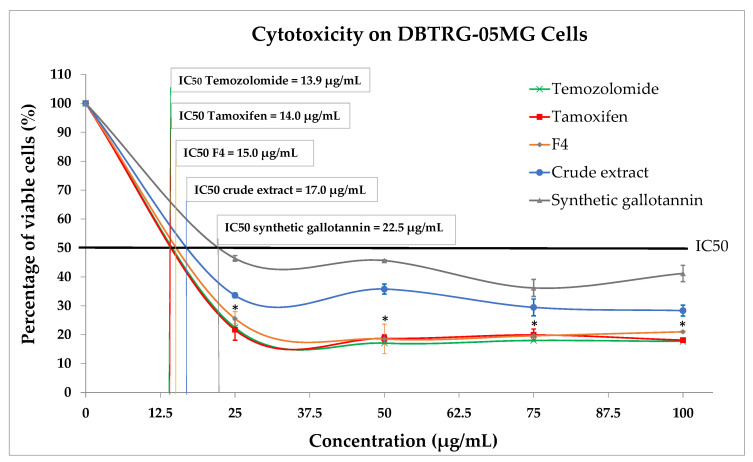
Cytotoxicity on human GBM cell line (DBTRG-05MG) after treatment with F4, crude extract, synthetic gallotannin and clinical cancer drugs (Temozolomide and Tamoxifen). IC_50_ = half maximal inhibitory concentration (* *p* < 0.05 for F4 vs. crude extract and synthetic gallotannin).

The cytotoxicity effect of F4 on the human GBM cancer cell line (DBTRG-05MG) was evaluated using the MTT assay and was compared to the effects of QI gall crude extract, synthetic gallotannin and clinically used chemo-drugs (Temozolomide and Tamoxifen). Generally, the cell viability was reduced in all treated groups, including the F4 (Figure 8). Nonetheless, F4 significantly inhibited GBM cells more effectively (IC_50_ = 15.0 µg/mL) compared to the QI gall crude extract (IC_50_ = 17.0 µg/mL) and synthetic gallotannin (IC_50_ = 22.5 µg/mL). This result is expected due to the significantly higher antioxidant potential possessed by F4, compared to the QI gall crude extract and synthetic gallotannin. Moreover, it was found that the cytotoxicity potential exerted by F4 (IC_50_ = 15.0 µg/mL) was similar to Temozolomide and Tamoxifen, in which there was no significant difference between the inhibitory effects exerted by F4 as compared to Tamoxifen (IC_50_ = 14.0 µg/mL) and Temozolomide (IC_50_ = 13.9 µg/mL). These close similarities between F4 and clinical anticancer drugs suggest that F4 could be a potential alternative anti-GBM agent other than the currently used chemo-drugs.

## 3. Discussion

This is the first report of the development of a two-phase extraction process to enrich a natural phenol compound known as gallotannin from the galls of the QI medicinal plant. Moreover, the gallotannin-enriched fraction obtained from this study was found to be more effective in combating GBM cancerous cells in vitro as compared to its crude extract, as well as the synthetic pure gallotannin available commercially. The effect of this enriched fraction also was found comparable to the effect of two clinically used chemo-drugs (Temozolomide and Tamoxifen) for in vitro GBM treatment.

GBM is one of the most aggressive brain cancers, with a low prognosis rate of an average of 14–15 months survival after diagnosis, making it a crucial public health issue [15]. Currently, GBM patients depend on trio-strategies consisting of surgery, radiotherapy and chemotherapy for treatment. However, due to several barriers posed by the anatomy and physiology of the brain, the effectiveness of these conventional cancer treatments is very much restricted, particularly for treating malignant brain cancer such as GBM [15]. Therefore, the development of an alternative treatment for GBM using the natural anticancer compound derived from the QI plant was explored in this study.

Natural bioactive compounds present in the QI plant have been found useful as remedies for various diseases since ancient times, and they are now becoming an important research area for novel drug discovery, especially for cancer and infectious diseases [16]. In this study, an optimized extraction protocol to isolate the targeted anticancer compound (gallotannin) from QI plant materials was carefully developed. Firstly, the crude extract of QI galls was prepared using soxhlet extraction with a water solvent at boiling temperature to obtain an aqueous extract containing water-soluble compounds. A pure water solvent was selected because herbal medicines are traditionally prepared by boiling the herbs in water. Furthermore, it was previously demonstrated that the QI gall aqueous extract contained high amounts of gallotannin [10]. Subsequently, in order to further enrich the gallotannin from the crude extract for specific biological assessment, the crude extract was fractionated using an open-column adsorption chromatography packed with Diaion HP-20, a non-polar styrene-divynilbenzene copolymer adsorbent resin. It was previously demonstrated that Diaion HP-20 resin is an effective adsorbent to eliminate trace elements, heavy metal ions and impurities to generate pure extracts [17]. This finding is in agreement with our study, in which an extra unknown compound was detected only in the QI crude extract (before fractionation) but not in the fractionated extracts, as evidenced by TLC and HPLC analysis. These data suggested that the fractionation procedure using Diaion HP-20 described in this study had successfully removed non-targeted compounds from the crude extract, generating a purer extract after fractionation.

Besides playing a crucial role to remove unknown impurities, Diaion HP-20 resin also provides a stationary support for adsorption and desorption of targeted compound(s) for preparative separation/purification. The capacity of adsorption and desorption (also known as binding–elution performance) of phenolic compounds such as gallotannin is highly dependent on the polarity differential between the resin (stationary phase) and the eluent solvent (mobile phase). In this study, the non-polar Diaion HP-20 column was eluted using a water (highly polar) and methanol (semi-polar) dual-solvent at gradient ratios to shift the polarity of the resin with the change of the eluent polarity. A report has demonstrated that solvents with different polarity indexes could significantly enrich phenolic compounds into different fractions based on the polarity of the compound [18]. This is consistent with our screening data, in which the concentration of gallotannin was distinctly different in each different elution, with the highest concentration retained in the 50% methanol eluent (F4). This separation is due to the law of similarity and inter-miscibility, which states that a solute is more miscible in a solvent with a similar polarity value [19].

The choice of solvent for extraction and fractionation is crucial for the overall success of natural product isolation. Besides choosing the solvent type which can yield a higher percentage of the desired compound, considerations must be taken to assure that the biological activities of plant constituents are not lost, distorted or destroyed during the preparation of the extract from the plant materials using the selected solvent [20].. Therefore, the DPPH assay and reducing power assay were used in this study to investigate the antioxidant potential of the QI gall extract before and after fractionation. For both assays, a commercially available pure synthetic gallotannin was also included to compare the biological activity of natural vs. synthetic gallotannin. To our surprise, the selected fraction with the highest gallotannin concentration (F4) was found to exhibit significantly higher antioxidant activity, not only compared to its crude extract, but also compared to the pure synthetic gallotannin. This could be due to the synergistic effects of other phenolic compounds such as the gallic acid and polyphenols present in the QI galls that share a similar polarity as the gallotannin [11]. These compounds may be present in the F4 extract and aid in radical scavenging activity, making F4 a better antioxidant agent drug as compared to the single pure gallotannin compound. Our finding is in accordance with the previous report, which stated that all the natural products exist as a complex mixture with other compounds and the biological activities of a plant are usually modulated by a set of related compounds produced by the plant. Despite that these active compounds can be purified for single drug development, they are sometimes better to exert certain biological properties when combined with other natural compounds due to synergic effects [21].

Moreover, F4 also exerted a significantly higher inhibitory effect on cancerous GBM cells when compared to its crude extract and synthetic gallotannin. The fascinating inhibitory effects of this gallotannin-enriched F4 on GBM cells could be related to its high antioxidant activity. Observations from clinical, animal and in vitro studies showed that antioxidants have superior potential of ameliorating chemotherapeutic-induced toxicity [22]. Moreover, antioxidant supplementation during chemotherapy also successfully increased patient survival times [22]. Therefore, here, we postulate that the bioactive activity triggered by the F4 on GBM was significantly enhanced due to the significant increase of gallotannin and antioxidant properties in the F4.

In addition, we also found that the inhibitory effect exerted by F4 was similar to the clinically used chemo-drugs, Temozolomide and Tamoxifen, suggesting its potential as an alternative therapeutic agent for GBM treatment. However, we suggest that the mechanism of gallotannin-enriched F4 to inhibit malignant tumor growth could be different than the chemo-drugs because there was no significant antioxidant property detected in these two chemo-drugs. Our hypothesis is supported by a previous study which reported that Temozolomide is a DNA alkylating agent which induces cancerous cell apoptosis by arresting the cell cycle at G2/M [23], while Tamoxifen induces glioma cell apoptosis through its direct action on mitochondrial complex I inhibition [24]. Both of these chemo-drugs induce cancer cell death using different mechanisms than the F4, which is a natural phenolic compound with high antioxidant properties. Nonetheless, this study is limited to the effects observed on the selected human glioma cell line (DBTRG-05MG). We suggest further detailed studies on the mechanism of gallotannin-enriched F4 to inhibit malignant tumor growth in several different human glioma cell lines of different degrees of malignancy and grading to validate the efficacy of this compound before its application as a clinical therapeutic agent for GBM.

## 4. Materials and Methods

### 4.1. Plant Material Collection and Authentication

QI galls were purchased from a Chinese medicine store in Kelantan, Malaysia. The source and identity of the QI galls were verified by a qualified Traditional Chinese Medicine practitioner in the store. For further scientific verification, the plant material was sent to the International Islamic University Malaysia (IIUM) Herbarium Centre, Pahang, Malaysia, for validation. The verified voucher specimens (PIIUM 0229-2) were deposited in the IIUM Herbarium Centre (Supplementary Figure S1). The plant material was kept in a dry storage area at room temperature with good ventilation to control humidity and prevent the growth of fungus until usage.

### 4.2. Plant Extraction and Fractionation

Gallotannin was extracted from the QI galls based on our previously described methodologies with modifications [10,21]. In brief, 50 g of QI gall powder was weighed and placed in a medium-sized thimble. The thimble was put in a soxhlet extractor with 450 mL of distilled water and the extraction was carried out at water boiling point (~100 °C) for 6 h. After extraction, the aqueous crude extract was subjected to a rotary evaporator (Buchi AG, Flavil, Switzerland) at 50 °C to remove the excess water content and then freeze-dried into powder form. Then, 7 g of the QI crude aqueous extract powder was weighted and suspended in 5 mL of distilled water. A resin chromatography column was prepared using Diaion^®^ HP-20 resin (Mitsubishi Chemical Corporation, Tokyo, Japan). The column was rinsed with distilled water to remove any resin residue in the column before the loading of the sample. Then, the column was eluted with 0%, 10%, 25%, 50%, 75% and 100% methanol respectively, to obtain six fractions (F1–F6). The fraction samples were collected and dried using a rotary evaporator. TLC and HPLC assays were performed to detect and quantify gallotannin in each fraction.

### 4.3. Thin-Layer Chromatography (TLC) Analysis

Aluminum sheets coated with silica gel 60 F254 were used to perform TLC separation (Merck Kga A, Darmstadt, Germany): 1 mg/mL of QI crude aqueous extract and fractionated extracts were prepared and spotted on the TLC plate, and 1 mg/mL of synthetic gallotannin (Sigma, Burlington, MA, USA) also was spotted on the same TLC plate as a positive control. The spotted TLC was left to dry and put into a developing chamber with chloroform:methanol:water (12:4:0.1). The TLC plate was removed once the solvent of the developing chamber almost reached the top of the plate. Then, the plate was immediately viewed under short wavelength ultraviolet (UV) light (254 nm). The presence of bands on the TLC plate was observed and recorded. The retention factor (Rf) value of each band was determined using the following formula:

$$\mathrm{Retention}\;\mathrm{factor}\;\left(\mathrm{Rf}\right)=\frac{\mathrm{Distance}\;\mathrm{travelled}\;\mathrm{by}\;\mathrm{the}\;\mathrm{compound}}{\mathrm{Distance}\;\mathrm{travelled}\;\mathrm{by}\;\mathrm{the}\;\mathrm{solvent}\;\mathrm{front}}$$

### 4.4. High-Performance Liquid Chromatography (HPLC) Analysis

The concentration of the gallotannin active compound in the QI crude aqueous extract and all six fractions were analyzed using HPLC, and 100 µg of samples were dissolved in 1 mL of methanol for each. Then, the mixture was filtered using a 0.2 µm nylon filter membrane (Bioflow, Kuala Lumpur, Malaysia) and injected into a HPLC system consisting of a Nexera XR LC-20 AD XR Compact LC System, SPD-M20A Photodiode Array Detectors (DAD), Prominence SIL-20A/AC Autosamplers, CBM-20A Communication Bus Module and a Prominence CTO-20A Column oven. A Shim-pack GIST C18 HPLC Packed column of 250 × 4.6 mm (Shimadzu, Kyoto, Japan) was used as a separation column. Elution was carried out with solvent A (composed of 1% acetic acid) and solvent B (composed of 100% methanol) in gradient concentrations, starting with 95% solvent A and ending with 5% solvent B. The temperature was maintained at ambient temperature and the flow rate was maintained at 1 mL/min throughout the process. Synthetic gallotannin at gradient concentrations was used as a standard marker in this assay. Full UV spectra were recorded from 200 to 364 nm, and finally, the peak was identified at 280 nm. By using the calibration curve obtained from the standard gallotannin marker, the concentrations of gallotannin content in all fractionated samples were determined.

### 4.5. Antioxidant Activity

#### 4.5.1. DPPH Free Radical Scavenging Activity

Antioxidant potential of the selected QI gall fraction, crude aqueous extract, synthetic gallotannin and chemo-drugs (Temozolomide and Tamoxifen) was determined using the DPPH (2,2-diphenyl-1-picryl-hydrazyl-hydrate) free radical scavenging assay. Blank solution was used as a control. The samples were serially diluted with methanol to 0, 20, 40, 60, 80 and 100 µg/mL and added to 1 mL of 0.1 mM DPPH (Merck, Kenilworth, NJ, USA), respectively. The mixture was incubated in the dark at room temperature for 30 min. During the incubation period, antioxidant compounds in samples reduced the in situ formed DPPH radical into DPPHH, resulting in a change of color from purple to yellow, which had a maximum absorption wavelength at 517 nm. Therefore, the optical density (OD) of the sample was measured at 517 nm. The formula used to calculate the free radical scavenging activity was as follows:

$$\mathrm{Radical}\;\mathrm{Scavenging}\;\mathrm{Activity}=\frac{\mathrm{OD}\;\mathrm{Control}-\mathrm{OD}\;\mathrm{Sample}}{\mathrm{OD}\;\mathrm{Control}}\times 100$$

#### 4.5.2. Reducing Power Assay

The ferric iron (Fe^3+^) reducing power of the QI gall extract before (crude extract) and after fractionation (F4) was determined by a method described by Mukherjee et al. [25] with slight modifications. Besides that, synthetic gallotannin and chemo-drugs (Temozolomide and Tamoxifen) were also included in the assay. All samples were dissolved in 1 mL of distilled water to prepare the sample solution at 100 µg/mL. Blank solution was prepared without adding any extract. Then, each of these samples were mixed with 2.5 mL of phosphate buffer (0.2 M, pH 6.6) and 2.5 mL of 0.1% (*w*/*v*) potassium hexacyanoferrate (K_3_Fe(CN)_6_). The mixtures were incubated in a water bath at 50 °C for 20 min. The reaction was arrested by adding 2.5 mL of 10% trichloroacetic acid (TCA) solution and then centrifuged at 3000 rpm for 10 min. After centrifugation, 2.5 mL of the upper portion of the supernatant was transferred into another 15 mL tube and mixed with 2.5 mL of distilled water and 0.5 mL of freshly prepared 0.1% (*w*/*v*) ferric chloride (FeCl_3_) solution. The mixture was incubated for 10 min at room temperature for color development. In this assay, the presence of antioxidants in the extracts resulted in a reduction of the ferric cyanide complex (Fe^3+^) to the ferrous cyanide form (Fe^2+^), thereby changing the solution into various shades from green to blue. Stronger reducing agents formed darker shades of blue that were maximally absorbed at 700 nm. Therefore, the OD absorbance values were measured at 700 nm. A higher absorbance of the reaction mixture indicated greater reducing power. The formula used to calculate the reducing power was as follows:

$$\mathrm{Reducing}\;\mathrm{power}=\frac{\mathrm{OD}\;\mathrm{Sample}}{\mathrm{OD}\;\mathrm{Control}}\times 100$$

### 4.6. Human Glioblastoma Cancer Cell Line Culture

The human glioblastoma cancer DBTRG-05MG cell line (ATCC CRL-2020) was purchased from American Type Culture Collection (ATCC, Manassas, VA, USA). The cell lines were cultured in a complete growth medium Roswell Park Memorial Institute (RPMI) 1640 (GIBCO, Waltham, MA, USA), supplemented with 10% fetal bovine serum (FBS) (GIBCO, Waltham, MA, USA) and 1% Penicillin–Streptomycin (GIBCO, Waltham, MA, USA). The cells were cultured until confluence in a humidified incubator under 5% CO_2_, at 37 °C.

### 4.7. Inhibitory Effects on Human Glioblastoma Cancer Cells

The DBTRG-05MG was harvested and plated on a 96-well plate with a density of 35,000 cells per well. The QI crude aqueous extract and selected QI fractions were added to the cells at the concentrations of 0, 2, 4, 6, 8 and 10 µg/mL respectively, and 1% DMSO was used as a negative control. Clinical drugs for cancer (Temozolomide and Tamoxifen) and synthetic gallotannin were used as positive controls. After 72 h of incubation, 10 µL of MTT (5 mg/mL of 3-(4,5-dimethylthiazol-2-yl)-2,5-diphenyltetrazolium bromide) solution was added to each well and incubated for another 4 h at 37 °C in the dark. The medium was discarded after 4 h of incubation time and 100 µL of DMSO was added to dilute the crystallization of MTT. The absorbance OD at 570 nm was measured using a microplate reader (Bio-Rad, Hercules, California, USA). The viable cells’ percentage was determined by the following formula:

$$\mathrm{Cell}\;\mathrm{Viability}\;\mathrm{Percentage}\left(\%\right)=\frac{\mathrm{OD}\;\mathrm{Sample}}{\mathrm{OD}\;\mathrm{Control}}\times 100$$

## 5. Conclusions

In summary, the present study investigated the potential therapeutic action of a gallotannin-enriched fraction (F4) from the QI gall crude aqueous extract for the most notorious brain cancer, GBM. Our study demonstrated that F4 significantly increased the radical scavenging activity compared to the crude extract and the synthetic pure compound. In addition, F4 also exhibited a similar inhibitory effect on GBM when compared to the currently used chemo-drugs for the treatment of GBM patients. We propose that the cytotoxic effects of F4 on GBM occur through the stimulation of antioxidant activity and suppression of oxidative stress, supported by its high antioxidant properties. However, further detailed studies on the pharmacokinetics, bioavailability, efficacy and the immediate and long-term side effects of F4 are required to draw a solid conclusion that this compound is capable as a therapeutic agent for GBM, comparable to chemo-drugs in clinical practice.

## Figures and Tables

**Table 1:** Fractions obtained from QI aqueous crude extract and the total dry weight and yield percentage for each fraction.

Initial Material	Fraction (F) Obtained	Yield	
		Total Dry Weight (g)	Percentage (%)
7 g of QI aqueous crude extract	F1 (0% methanol)	2.24	32
	F2 (10% methanol)	0.49	7
	F3 (25% methanol)	0.63	9
	F4 (50% methanol)	2.59	37
	F5 (75% methanol)	0.98	14
	F6 (100% methanol)	0.07	1
	TOTAL	7 g	100%

**Table 2:** Total concentration and recovery percentage of the gallotannin compound in the fractionated QI extract (F1 to F6).

Initial Material (100 µg/mL)	Gallotannin Yield	
	Concentration (µg/mL)	Recovery Percentage (%)
F1	5.61 ± 1.22	5.61
F2	7.08 ± 0.88	7.08
F3	37.44 ± 2.98	37.44
F4	71.15 ± 3.21	71.15
F5	47.06 ± 1.64	47.06
F6	3.95 ± 1.05	3.95

## Data Availability

Data is contained within the article and supplementary material.

## Supplementary Materials

The following are available online at https://www.mdpi.com/article/10.3390/plants10122581/s1, Figure S1: Verified voucher of QI gall specimen (PIIUM 0229-2) by International Islamic University Malaysia (IIUM) Herbarium Centre, Pahang, Malaysia.
