# Supplementary material for: Gallotannin-Enriched Fraction from Quercus infectoria Galls as an Antioxidant and Inhibitory Agent against Human Glioblastoma Multiforme
Source: Plants (Basel). 2021 Nov 25;10(12):2581. doi: 10.3390/plants10122581 (PMC8708140; doi:10.3390/plants10122581)
Supplement: Supplementary file 1 [file plants-10-02581-s001.zip › plants-1452414-supplementary.pdf]

Reference: IIUM/308/15/2/1/NMPC19-1/16
Date: 10th October 2019

Norhazilah Muhamad
Pusat Pengajian Sains Kesihatan
Universiti Sains Malaysia
Kampus Kesihatan
16150 Kubang Kerian
Kelantan

UKMB
VERIFIED
24 OCT 2019
Universiti Kebangsaan Malaysia
Herbarium

Dear Sr.,

Assalamualaikum Wrt.

PLANT SPECIES IDENTIFICATION

Kindly be informed that the plant specimen submitted for identification is confirmed as follows:

Voucher No.	Family Name	Scientific Name	Local Name
**PIIUM 0229-2**	**Fagaceae**	***Quercus infectoria*** **G.Olivier. (The tree species from which the gall is formed)**	**Manjakani (The gall)**

Student's name : Norhazilah Muhamad
Supervisor's name : Dr. Tan Suat Cheng
Herbarium : Kulliyyah of Pharmacy
Person in-charged : Asst. Prof. Dr. Norazian Mohd Hassan
Identified by : Dr. Shamsul Khamis

It should be noted that we are not involved with anything as a result of research conducted by the host.

Thank you and wassalam.

Sincerely,

ASST. PROF. DR. NORAZIAN BINTI MOHD HASSAN
Kulliyyah of Pharmacy
International Islamic University Malaysia
Jalan Sultan Ahmad Shah
Bandar Indera Mahkota
25200 Kuantan
Pahang

DR. SHAMSUL KHAMIS
Botanist
Herbarium UKMB
Faculty of Science and Technology
The National University of Malaysia (UKM)
43600 Bangi, Selangor

Voucher specimen no.: PIIUM 0229-2

No.	Type of information and details
1.	Family name of the specimen<br>**Fagaceae [1]**
2.	Scientific name of the specimen, (including the authority – if any)<br>***Quercus infectoria* G.Olivier [1]**
3.	Local name<br>**Manjakani (English common name/s: Oak galls, Magic nuts) [2,3]**
4.	Collector's name/s (with e-mail address if any)<br>**Norhazilah Muhamad (norhazilah@yahoo.com)**
5.	Student's name, matric no. & Institution<br>**Norhazilah Muhamad, P-SKD0001/16(R) & PPSK, USM Kelantan**
6.	Supervisor's name (with title)<br>**Dr Tan Suat Cheng**
7.	Date of collection<br>**24 July 2019**
8.	Plant part/s<br>**Gall (UP)[VS-3]**
9.	Locality where the plant was collected, (including latitude and longitude – if any<br>**Purchased from Kedai Ubat Heng De, Kota Bharu, Kelantan**
10.	Name of the person who determined the identification<br>**Dr. Shamsul Khamis**
11.	Altitude<br>**From sea level to c.1,700 m. [4]**
12.	Habitat or type of plant community<br>**Mixed scrub and deciduous woodland. Also found co-existing with other species of Oak in vegetation known as longos forests with abundant marsh areas dominated by arboreal Oak species. [4]**
13.	Habit<br>**The galls formed on the young twigs of *Q. infectoria* tree, as a result of the deposition of the eggs of the gall-wasp *Adleriagallae-tinctoriae*. [3]**
14.	Any other details about the plant that may be important<br>[Example: flower colour, soil type, slope, aspect, plant height and width, associated species, bark colour and type, sap (eg. milky, resinous, etc.), distinctive odour, dioecy, abundance, pollinators, herbivory, etc.]<br>**Galls are globular in shape, 10 to 25 mm in diameter. They have a short, basal stalk and numerous rounded projections on the surface. The galls are hard and heavy, usually sinking in water. Bluish- or olive-green in colour. [3]**

Verification date: 24 October 2019

15.	Plant/sample photos (various plant parts, preferably original photos)<br>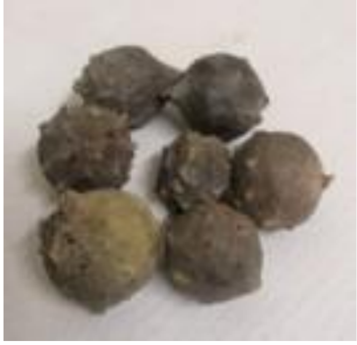 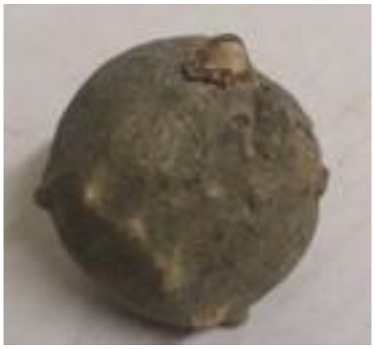<br>**The galls from *Q. infectoria***
16.	References<br>**1] http://www.theplantlist.org/tpl1.1/record/kew-173331**<br>**[2] http://www.globinmed.com/index.php?option=com_content&view=article&id=105888:quercus-infectoria-g-olivier&catid=286&Itemid=357**<br>**[3] Abdul Haque, A. S.; Ahmad, W.; -cology of *Quercus infectoria* Olivier – Galls: A Review. *Hippocratic Journal of Unani Medicine*, *11*(3), 105-118.**<br>**[4] https://www.iucnredlist.org/species/194176/56059946#habitat-ecology**
